# Phospholipase D activation is required for 1-aminocyclopropane 1-carboxylic acid signaling during sexual reproduction in the marine red alga *Neopyropia yezoensis* (Rhodophyta)

**DOI:** 10.1186/s12870-022-03575-z

**Published:** 2022-04-08

**Authors:** Toshiki Uji, Takuya Kandori, Shiho Konishi, Hiroyuki Mizuta

**Affiliations:** Laboratory of Aquaculture Genetics and Genomics, Division of Marine Life Science, Faculty of Fisheries Sciences, Hokkaido University, Hakodate, 041-8611 Japan

**Keywords:** *Neopyropia*, Red algae, Sexual reproduction, 1-aminocylopropane-1-carboxylic acid, Phospholipase D, Phosphatidic acid, Plant hormone

## Abstract

**Background:**

1-aminocyclopropane 1-carboxylic acid (ACC) is the immediate precursor of the plant hormone ethylene. However, recent studies have suggested that ACC also acts as a signaling molecule to regulate development and growth independently from ethylene biosynthesis. In red algae, ACC stimulates the switch from a vegetative to a sexual reproductive phase. However, despite evidence that ACC signaling in plants and algae is widespread, the mechanistic basis of the ACC signaling pathway remains unknown.

**Results:**

We demonstrate that exogenous ACC increased the activity of phospholipase D (PLD) and induced the accumulation of PLD transcripts in the marine red alga *Neopyropia yezoensis*. The product of PLD, the lipid second messenger phosphatidic acid (PA), also increased in response to ACC. Furthermore, the pharmacological inhibition of PLD by 1-butanol blocked ACC-induced spermatangia and carpospore production, but the inactive isomer t-butanol did not. In addition, 1-butanol prevented ACC-induced growth inhibition and inhibited transcript accumulation of genes upregulated by ACC, including extracellular matrix (ECM)-related genes, and alleviated the transcriptional decrease of genes downregulated by ACC, including photosynthesis-related genes.

**Conclusions:**

These results indicate that PLD is a positive regulator of sexual cell differentiation and a negative regulator of growth. This study demonstrates that PLD and its product, PA, are components of ACC signaling during sexual reproduction in *N. yezoensis*.

**Supplementary Information:**

The online version contains supplementary material available at 10.1186/s12870-022-03575-z.

## Background

1-aminocylopropane-1-carboxylic acid (ACC) is known as the direct precursor of the plant hormone ethylene, which regulates a wide range of developmental processes and stress responses [1, 2]. Exogenous ACC application has been used as a proxy for ethylene in numerous experiments over decades of research on ethylene-associated signaling. However, there is emerging evidence from the model plant *Arabidopsis thaliana* that ACC itself may have a signaling role that is distinct from its role as a precursor of ethylene [3, 4]. For example, an acs octuple mutant with deficient ACC production exhibited a remarkable decrease in a seed set that was not observed in ethylene-insensitive mutants [5]. Other studies have uncovered that ACC signaling independent of the influence of ethylene can regulate vegetative processes such as early root and hypocotyl elongation in seedlings [6] and cell wall function [7]. In addition, recent studies suggest that ACC functions independently of ethylene in the basal land plant *Marchantia polymorpha *[8, 9]. Given the apparent significance of ACC signaling, elucidating the mechanistic basis of ACC signaling pathways is an important goal of contemporary research.

Phospholipase D (PLD) hydrolyzes membrane lipids, producing phosphatidic acid (PA) and free hydrophilic head groups, such as choline or ethanolamine [10]. PA is now regarded as a lipid signaling molecule that regulates numerous physiological processes in eukaryotes [11, 12]. In higher plants, PLD and its product PA mediate the signaling of various plant hormones, including abscisic acid (ABA), ethylene, jasmonic acid, and salicylic acid (SA) [10, 13]. To date, 1-butanol, an antagonist of PLD-dependent PA production, has been used to investigate the function of PLDs in plant hormone signaling. For example, one study of ABA and gibberellin (GA) signaling in barley aleurone tissue found that both pathways were inhibited by 1-butanol [14]. In addition, 1-butanol has also been found to block SA-induced stomatal movement in guard cells in *A. thaliana* [15].

The red alga *Pyropia/Neopyropia* (formerly *Porphyra*), which belongs to the Bangiales, is a significant marine crop that is harvested to produce nori, an edible seaweed commonly used to wrap sushi and onigiri. During the sexual life cycle of *Pyropia/Neopyropia*, the blade gametophytes bear non-flagellated male (spermatia) and female (carpogonia) gametes on the gametophytes. Fertilization occurs when the female gametes are retained on the gametophyte, and successive cell divisions produce clones of the zygote. It is the carpospores that then grow into filamentous sporophytes. Recent research from our group has demonstrated that the application of ACC induced gametogenesis and growth suppression in the monoecious species *Neopyropia yezoensis* and the dioecious species *Pyropia pseudolinearis* [16, 17]. Exogenous ACC analogs also promoted sexual reproduction in the same manner as ACC, whereas ethephon, an ethylene-releasing compound, did not stimulate sexual reproduction in *N. yezoensis* [18, 19]. In RNA-seq, transcripts associated with cell division, vesicular-trafficking, and extracellular matrix (ECM) were found to be up-regulated in gametophytes treated with ACC, while transcripts involved in translation, plastid transcription and photosynthesis were downregulated [16, 17]. In addition, the application of ACC generated reactive oxygen species (ROS) mediated by NADPH oxidase activity, increased ascorbate (AsA) synthesis, and decreased glutathione (GSH) synthesis [18]. Taken together, these results suggest that ACC may play a role as a signaling molecule independent from its role in ethylene signaling and that it may be involved in the regulation of sexual reproduction through changing the redox state of *Pyropia/Neopyropia*.

To date, several studies have suggested that PLD is involved in development processes and abiotic stress responses in *Neopyropia* species [20, 21]. In addition, the previous RNA-seq data [16] showed transcript accumulation of PLD gene upregulated by ACC. We therefore, investigated whether PLD and PA were required for the ACC response during sexual reproduction in *N. yezoensis*. We provide evidence that PLD and PA are required for the signal transduction events ultimately leading to ACC-induced sexual reproduction in *N. yezoensis*.

## Methods

### Algal materials and chemical treatments

Gametophytic blades of *N. yezoensis* strain TU-1 were cultured in a medium of sterile vitamin-free Provasoli’s enriched seawater (PES [22];) at 15 °C under cool-white fluorescent lamps at 40 μmol photons m^− 2^ s^− 1^ irradiance with a photoperiod regime of 10 h light:14 h dark. For PLD inhibitor experiments, we grew immature gametophytes (approximately 20 mm in blade length) that were microscopically determined to bear only vegetative cells in 90-mm-diameter petri dishes with 40 mL PES. Plates were then placed in a shaking incubator. Gametophytes were then treated with 0 or 50 μM ACC (Tokyo Chemical Industry, Tokyo, Japan) in the presence of 1-butanol (n-butyl alcohol) or t-butanol (tert-butyl alcohol) for 10 days. After 10 d, gametophytes that had formed spermatangia were observed under a Leica DM 5000 B microscope (Leica Microsystems, Tokyo, Japan), since the carpogonia of *N. yezoensis* are almost indistinguishable from surrounding vegetative cells. Formation of spermatangia in *N. yezoensis* generally begins in apical regions. Thus, in this study, gametophyte maturity level was evaluated using a four level scale as follows: “a”: an upper part without spermatangia, “b”: an upper part with spermatangia exhibiting slight discoloration, “c”: an upper part with spermatangia exhibiting clear discoloration, and “d”: an upper part with degraded cell walls and released spermata. In addition, after 14 d, the number of discharged carpospores attached to the bottom of the dishes was determined under a microscope as an index of the rate of formation of female gametes. Gametophyte blade lengths were also measured after 10 d, and the growth rate was calculated as the mean percentage of length increase per day using the following formula: Growth rate = [100(BLt − BL0)/BL0]/t, where BL0 = initial blade length, BLt = blade length as measured on day t of culture, and t = culture time in days.

### PLD activity assay

Phospholipase D (PLD) activity was determined using an Amplex™ Red Phospholipase D Assay Kit (Thermo Fisher Scientific K.K., Tokyo, Japan), according to the manufacturer’s protocol with minor modifications. For the PLD activity assay, vegetative gametophytes from blades approximately 20 mm in length (0.05 g fresh weight; FW) were cultured in a 100 mL culture medium containing 50 μM ACC for 0, 3, and 7d. Samples were then ground in liquid nitrogen with a pestle and mortar. For each sample, the resulting homogenate was added to 0.5 mL of 50 mM Tris-HCl buffer at pH 8.0 and centrifuged at 4 °C for 5 min at 15,000×g. Enzyme activity was detected by adding 100 μL of the supernatant to the working solution of the Amplex Red reagent. The fluorescence was detected using a spectrofluorometer (FB-750, Jasco, Tokyo, Japan) at an excitation wavelength of 545 nm and an emission wavelength of 610 nm to decrease interference from autofluorescence caused by photosynthetic pigments. The resulting fluorescence was subtracted from the background fluorescence derived from samples that did not contain the Amplex Red reagent working solution. Relative PLD activity was calculated as a ratio of the measurement taken at 0 d to that of another taken after ACC treatment. All data are presented as mean ± standard deviation (SD) of five biological replicates.

### PA measurement

Total PA content was measured by a coupled enzymatic reaction system using the Total Phosphatidic Acid Assay Kit (Cell Biolabs, Inc., San Diego, CA.). Experimental procedures were performed according to the manufacturer’s protocol with minor modifications. In brief, vegetative gametophytes from blades approximately 20 mm in length (0.05 g FW) were cultured in a 100 mL culture medium containing 50 μM ACC for 0, 3, and 7d. Samples were then ground in liquid nitrogen with a pestle and mortar. For each sample, the resulting homogenate was added to 0.75 mL methanol. Next, we added 1.15 mL 1 M NaCl and 1.25 mL chloroform to each sample and mixed the constituents thoroughly. After centrifugation at 4 °C for 10 min at 1500×g, the upper aqueous phase was discarded, and the lower chloroform phase was washed twice with a PEU solution, which was prepared by mixing 50 mL chloroform, 50 mL methanol, and 45 mL 1 M NaCl. Finally, the lower organic phase was dried under a gentle stream of nitrogen and dissolved in the provided Assay Buffer. Fluorescent signals were detected using a spectrofluorometer (FB-750, Jasco, Tokyo, Japan) at an excitation wavelength of 540 nm and an emission wavelength of 590 nm. Relative PLD production was quantified as a ratio of that PLD production at 0 d after ACC treatment. All data are presented as mean ± SD of five biological replicates.

### Bioinformatics analysis

Sequences of two *NyPLD* (*NyPLD1* and *NyPLD2*) genes were retrieved from *N. yezoensis* genome sequence data [23]. Additionally, one new *NyPLD* gene (*NyPLD3*) was identified in the genome sequence data (Accession ASM982973v1 in the NCBI GenBank Database). The conserved domains of NyPLD proteins were confirmed by analysis using the “SMART” protein architecture research tool (http://smart.embl-heidelberg.de/).

### Transcriptional analysis

RNA extraction and qRT-PCR were performed as described by [24]. Total RNA was extracted using an RNeasy Plant Mini Kit (Qiagen, Hilden, Germany) following the manufacturer’s instructions. Extracted RNA was purified using a TURBO DNA-free kit (Invitrogen/Life Technologies, Carlsbad, CA) to obtain DNA-free RNA. First strand cDNA was synthesized from 0.5 μg total RNA using the PrimeScript II First Strand cDNA Synthesis Kit (TaKaRa Bio, Shiga, Japan). cDNA was diluted 10-fold for qRT-PCR analysis; for each reaction, 1.0 μl of the diluted cDNA was used as a template in a 20 μL reaction volume containing KOD SYBR® qPCR Mix (TOYOBO, Osaka, Japan). qRT-PCR was then performed as per the manufacturer’s instructions using a LightCycler® 480 System (Roche Diagnostics, Basel, Switzerland) under the following conditions: 30 s at 95 °C followed by 40 cycles of 5 s at 95 °C and 31 s at 55 °C. mRNA levels were then calculated using the 2^−△△Ct^ method and were normalized to the observed expression level of 18S ribosomal RNA (*Py18SrRNA*) gene [16]. Relative expression levels over time were calculated as a ratio of the observed mRNA level at a given time point to the mRNA level present at 0 d after ACC treatment. qRT-PCR was performed in triplicate. Table S1 lists primers that were used for these analyses.

### Statistical analysis

All data were expressed as the mean ± SD. For the PLD activity, PA content, and growth rate experiments, Mann–Whitney U tests were performed to determine whether differences in mean were statistically significant. For all analyses, *p* < 0.05 (significant) or *p* < 0.01 (highly significant) were used as thresholds of statistical significance.

## Results

### ACC stimulates PLD activation and PA production

**Fig. 1:**
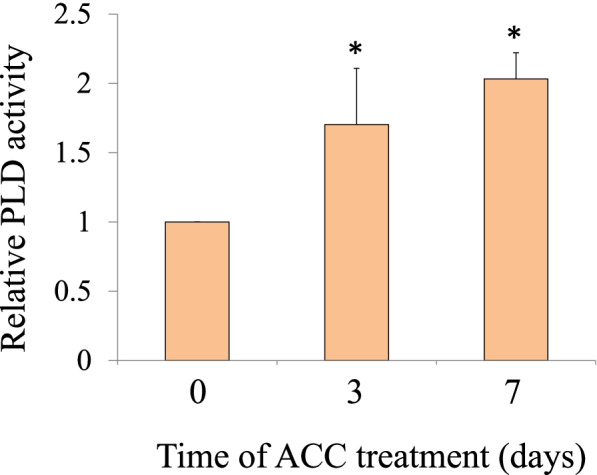
1-aminocylopropane-1-carboxylic acid (ACC)-stimulated PLD activity in *N. yezoensis*. Gametophytes were treated with ACC for 3, or 7d. Results are presented as measured relative activity compared to untreated gametophytes (0 d). Asterisks indicate a statistically significant difference at *P* < 0.05 between the control and the specified treatment. All data are presented as mean ± SD of five independent experiments

**Fig. 2:**
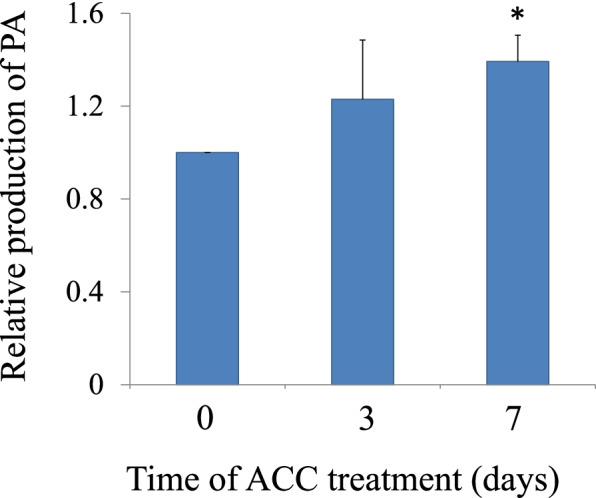
1-aminocylopropane-1-carboxylic acid (ACC) stimulated the production of phosphatidic acid (PA) in *N. yezoensis*. Gametophytes were treated with ACC for 3, or 7d. Results are presented as the relative amount compared to untreated gametophytes (0 d). Asterisks indicate a statistically significant difference at *P* < 0.05 between the control and the specified treatment. All data are presented as mean ± SD of five independent experiments

We measured PLD activity in response to ACC to determine the role played by PLD in ACC-activated signal transduction in *N. yezoensis*. Gametophytes that were cultured after 7d of ACC treatment formed spermatangia exhibiting slight or clear discoloration on the upper parts, but gametophytes treated with ACC for 3d did not. Gametophytes treated with ACC displayed increased PLD activity at 3 d after treatment (1.70-fold) with peak activity at 7 d (2.03-fold) (Fig. 1). Next, we examined the effects of ACC treatment on PA production during sexual reproduction. The PA content of gametophytes then increased (1.22-fold) 3 d after ACC treatment, and rose again at 7 d (1.39-fold) (Fig. 2). Taken together, these results indicate that PA production caused by ACC treatment is dependent on PLD activity.

### Characterization of PLD genes and their expression in response to ACC

**Fig. 3:**
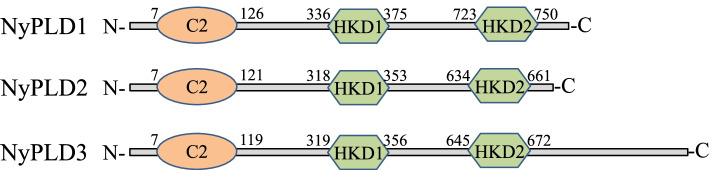
Schematic representations of the conserved domain and motifs of NyPLDs. Domains and motifs were determined by searching the NyPLD protein sequences using the SMART protein architecture tool. C2; Ca^2+^/phospholipid binding domain, HKD; conserved catalytic region. Numbers correspond to amino acid positions from the first methionine residue

In addition to our previous RNA-seq data, evidence of increased PLD activity and PA levels in gametophytes treated with ACC led us to characterize the PLD genes found in *N. yezoensis*. We identified three genes encoding PLDs in the *N. yezoensis* genome sequence (Table 1 and Fig. 3). NyPLD protein lengths ranged from 797 to 1201 amino acid residues, and BLAST analysis revealed that their protein sequences exhibited 79.5–90.4% identity with PLDs found in the red marine alga *Porphyra umbilicalis*. NyPLD proteins comprised highly conserved catalytic HKD motifs “HxKxxxxD” (HKD1 and HKD2) found at the C-terminal. Furthermore, we found C2 domains, which bind Ca^2+^ and other effectors including phospholipids and inositol phosphates [25], at the N-terminals of all NyPLDs. Given these characteristic domains, we identified these three NyPLDs as members of the C2-PLD family.

**Table 1:** The information of NyPLDs

Name	Contig ID	ORF (aa)	HKD1	HKD2
NyPLD1	contig_17468_g4270	881	**H**Q**K**TVVV**D**	**H**S**K**MAIF**D**
NyPLD2	contig_1916_g311	797	**H**S**K**AVVV**D**	**H**A**K**MMIV**D**
NyPLD3	g1.g7400	1201	**H**Q**K**TIIC**D**	**H**S**K**AVIF**D**

**Fig. 4:**
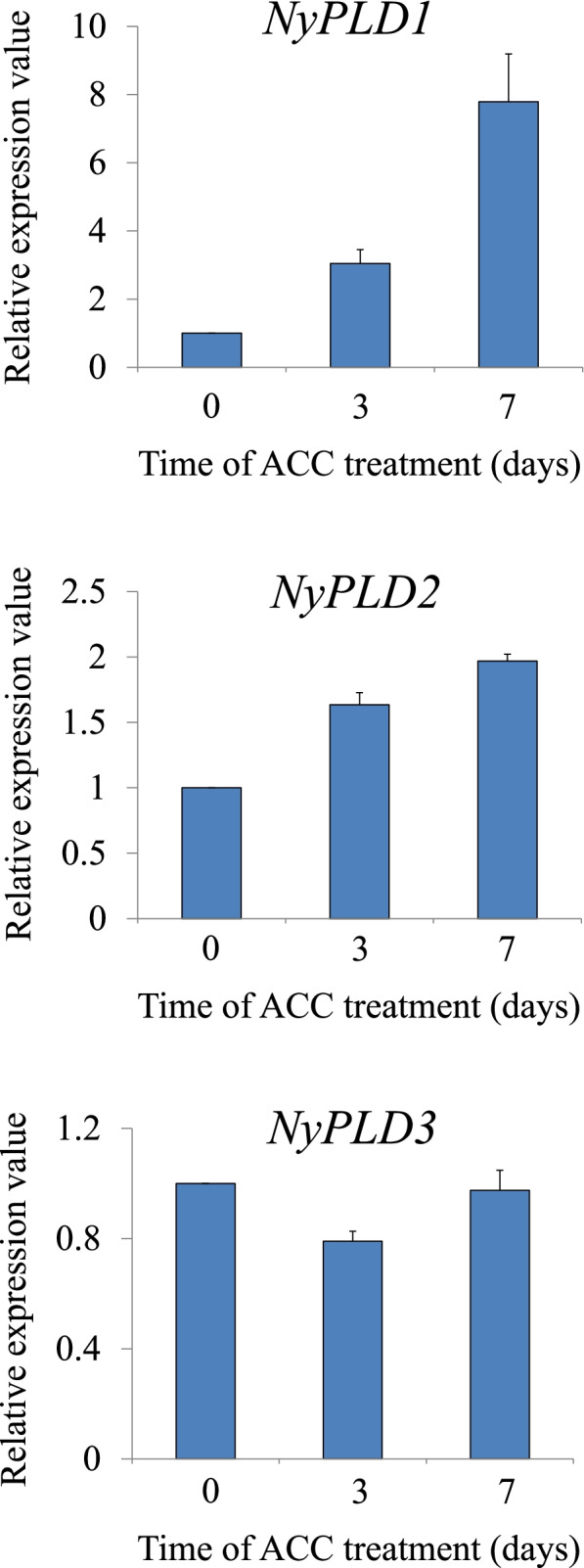
1-aminocylopropane-1-carboxylic acid (ACC)-activated PLD expression. RNA samples were prepared from gametophytes that were treated with 50 μM ACC for 3, or 7d, and expression levels were assessed using the *Ny18SrRNA* gene for normalization. Results are presented as relative expression compared to untreated gametophytes (0 d). All data are presented as mean ± SD of three independent experiments

Next, we analyzed the expression of *NyPLDs* in response to exogenous ACC treatment using real-time PCR (Fig. 4). We found that the mRNA transcript levels of *NyPLD1* had increased by 3 d after treatment (3.04-fold), and peaked at 7 d (7.79-fold). By contrast, the expression levels of *NyPLD2* and *NyPLD3* were only slightly upregulated and nearly unchanged, respectively, in response to ACC treatment. These results suggested that NyPLD1 is responsible for the increased PLD activity caused by ACC treatment.

### The PLD inhibitor 1-butanol blocks the ACC-induced PLD activity response

We next examined whether inhibition of PLD enzyme activity would block the induction of sexual reproduction caused by ACC treatment. PLDs can use primary alcohols as an acceptor of a phosphatidyl moiety instead of H_2_O, and in the presence of a primary alcohol such as 1-butanol, PLD catalyzes a transphosphatidylation reaction that leads to the formation of phosphatidylalcohol instead of PA [26]. Therefore, 1-butanol is generally used to inhibit PA formation by PLD. By contrast, tertiary alcohols such as t-butanol are not substrates of this transphosphatidylation reaction and can, therefore, be used as a control for any nonspecific butanol effect [26]. Moreover, we performed preliminary experiments that indicated that the application of 1-butanol in concentrations greater than 0.04% dramatically inhibited the growth of *N. yezoensis*. Thus, we treated vegetative gametophytes with 0.02% 1-butanol or t-butanol to assess whether their effects on ACC action differed.

**Fig. 5:**
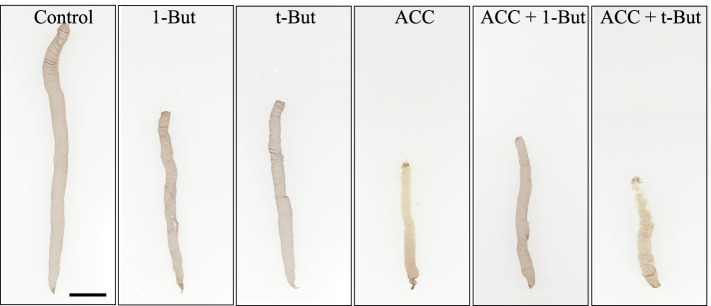
Effects of the PLD inhibitor 1-butanol in the presence of 1-aminocylopropane-1-carboxylic acid (ACC). Gametophytes were cultured in media containing 0 (Control) or 50 μM ACC (ACC). Co-treatments included a 10-day application of ACC with 0.02% 1-butanol (1-But + ACC), 0.02% t-butanol (t-But + ACC), 0.02% 1-butanol (1-But - ACC), and 0.02% t-butanol (t-But - ACC) alone for 10 d. We found that the thalli treated with ACC alone or by co-treatment with ACC and t-But formed many spermatangia in the upper regions and that these were often clear or discolored. Other thalli exposed to co-treatment with ACC and 1-But exhibited no discoloration in the upper regions. Scale bar = 10 mm

**Fig. 6:**
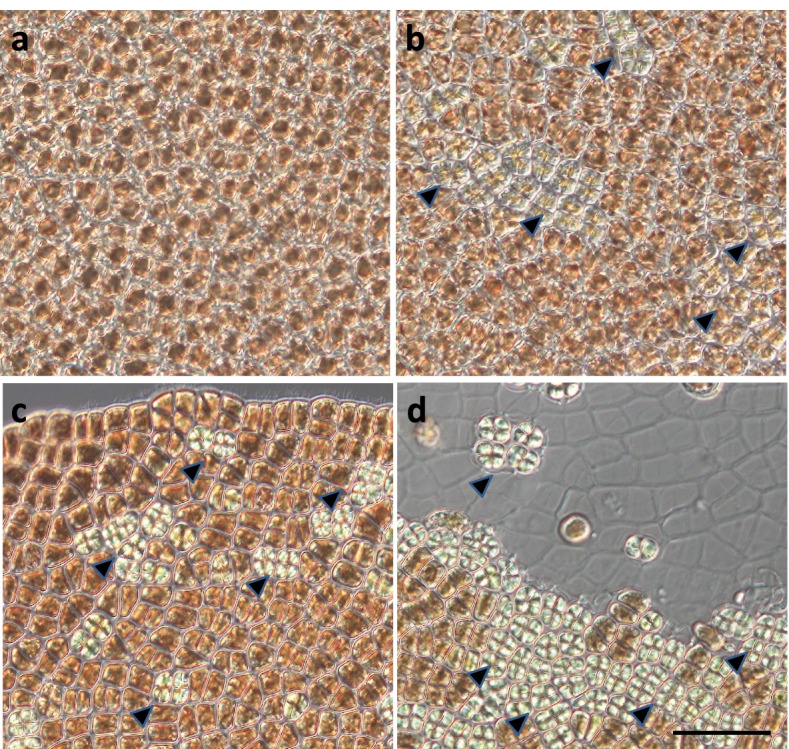
Mature levels of *N. yezoensis* for evaluation in the pharmacological experiments. a: the gametophyte without spermatangia, b: the gametophyte with spermatangia exhibited slight discoloration, c: the gametophyte with spermatangia exhibited clear discoloration, and d: the gametophyte degraded cell walls and released spermata. Arrowheads indicate spermatangia. Scale bar = 50 μm

**Fig. 7:**
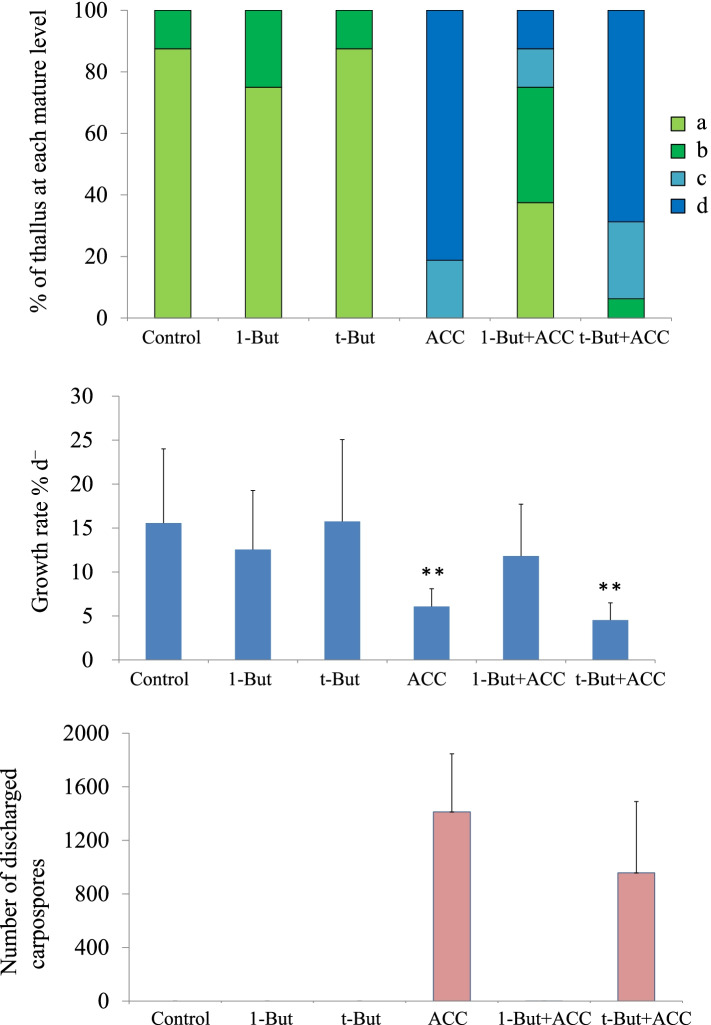
A PLD inhibitor blocks 1-aminocylopropane-1-carboxylic acid (ACC)-induced sexual reproduction. **A** Suppression of progression of maturity in the presence of the PLD inhibitor. Gametophytes were cultured in media containing 0 (Control) and 50 μM ACC (ACC) or a combined treatment of ACC and 0.02% 1-butanol (1-But + ACC), ACC and 0.02% t-butanol (t-But + ACC), 0.02% 1-butanol without ACC (1-But), or 0.02% t-butanol (t-But) alone. Plant maturity levels are illustrated as Fig. 6. Data were calculated in four independent experiments with four thalli for each condition (*n* = 16). **B** Alleviation of ACC-induced growth inhibition in the presence of the PLD inhibitor. Data are expressed as mean ± SD of four independent experiments with four thalli for each condition (*n* = 16). Asterisks indicate significant differences at *P* < 0.01 between the controls and treatments. **C** We also observed suppression of ACC-induced carpospore formation in the presence of the PLD inhibitor. The number of carpospores released from gametophytes was counted under microscope after 14d of culture. Data are expressed as means ± SD of two independent experiments with four thalli for each condition (*n* = 8)

We found that gametophytes treated with ACC produced colorless spermatangia on the upper parts of the thalli after 10 d of treatment; this was consistent with previous observations (Fig. 5). By contrast, the presence of 1-butanol inhibited ACC-induced spermatangia production, but the presence of the inactive t-butanol isomer did not (Fig. 5). In this study, we evaluated gametophyte maturity on a four-point scale described above (Fig. 6). When gametophytes were treated with a control or with butanol alone, all thalli (100%) were classified as “a” or “b” (Fig. 7A). By contrast, thalli treated with ACC alone (100%) or co-treated with ACC and t-butanol (93%) were classified as “c” or “d” (Fig. 7A). Moreover, when gametophytes were treated simultaneously with ACC and 1-butanol, most thalli (75%) were classified as “a” or “b” and showed suppression of the ACC-induced progression in maturity, likely caused by the 1-butanol treatment (Fig. 7A).

Next, we examined gametophyte growth rates. When gametophytes were cultured in media containing 50 μM ACC and without butanol, the growth rate was 6.0% d^− 1^, whereas untreated (control) gametophytes showed a growth rate of 15.5% d^− 1^ (Fig. 7B). However, we found that 1-butanol treatment prevented ACC-linked inhibition of growth (i.e., gametophytes treated showed a growth rate of 11.8% d^− 1^), whereas t-butanol did not show a significant effect on the inhibitory action of ACC (4.5% d^− 1^) (Fig. 7B). The mean number of carpospores released from the gametophytes treated with ACC was approximately 1412, whereas the mean of untreated control gametophytes was zero, and the mean of gametophytes co-treated with ACC and 1-butanol was approximately 1 (Fig. 7C). Taken together, these results indicate that the inactive t-butanol isomer had no inhibitory effect on ACC-induced carpospore production, in contrast to the significant effect of 1-butanol. Therefore, we conclude that treatment with 1-butanol significantly blocked the induction of sexual reproduction by exogenous ACC treatment in *N. yezoensis*.

**Fig. 8:**
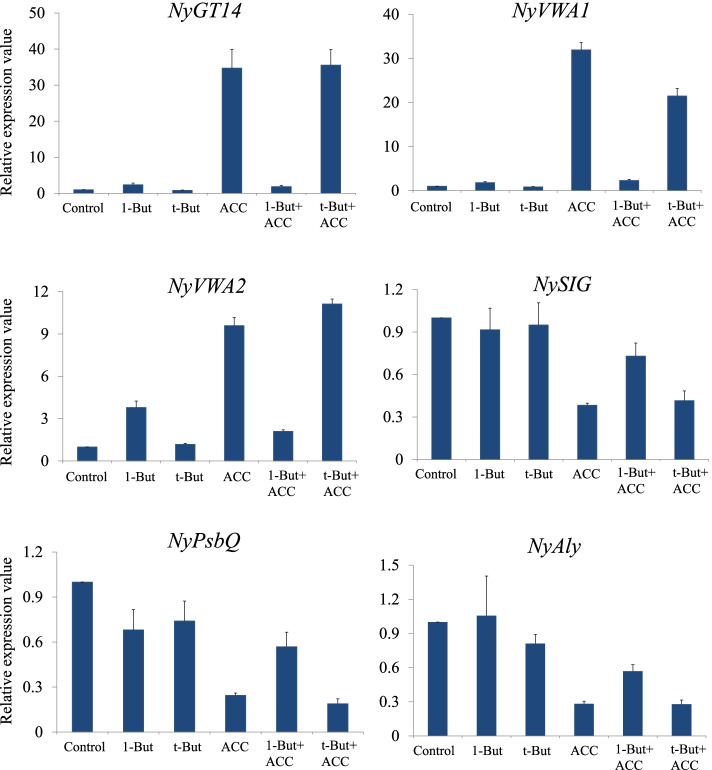
The PLD inhibitor affects the expression of ACC-responsive genes in *N. yezoensis*. RNA samples were prepared from gametophytes that were cultured in media containing 0 (Control) and 50 μM ACC (ACC) and from those containing co-applications with 0.02% 1-butanol (1-But + ACC), 0.02% t-butanol (t-But + ACC), 0.02% 1-butanol (1-But), or 0.02% t-butanol (t-But) alone for 3 d. Expression levels were determined using the *Ny18SrRNA* gene for normalization. *NyGT14*, *NyVWA1*, and *NyVWA2* were selected as representative genes that were upregulated by ACC, whereas *NySIG1*, *NyPsbQ*, and *NyAly* were selected as representative genes that were downregulated by ACC genes. These results are presented as relative expression level at the given time point relative to that present in untreated gametophytes (0 d). All data are presented as mean ± SD from three independent experiments

Finally, we examined the inhibitory effects of 1-butanol on the expression of ACC-responsive genes using qRT-PCR. Based on previous RNA-seq data [16], we selected three highly upregulated and three highly downregulated genes as representative ACC-responsive genes (Table S2). As illustrated in Fig. 8, the expression of ACC-upregulated genes including ECM-related genes, such as *NyGT14* and *NyVWA1*, strongly increased (31.96- to 34.73-fold, respectively) after 3 days of ACC treatment. However, the presence of 1-butanol strongly inhibited this increase in gene expression by treatment with ACC (we observed increases of only 1.88- to 2.31-fold, respectively). By contrast, the presence of t-butanol showed no effect on the expression levels of ACC-responsive genes. Moreover, the presence of 1-butanol alleviated the decrease in expression of genes that were downregulated by ACC, including photosynthesis-related genes, such as *NySIG1* and *NyPsbQ*, whereas t-butanol had no effect on the ACC-induced downregulation of these genes.

## Discussion

Recent studies have proposed that in land plants and red algae, the ethylene precursor ACC may play a signaling role that is independent of the ethylene signaling pathway [4]. However, little is known regarding the mechanisms involved in regulating ACC signaling. In this study, we demonstrated that PLD activation is required for ACC signaling during sexual reproduction of *N. yezoensis*. This report is the first to describe the link between PLD and plant hormone signaling in red algae. In land plants, PLD has been thought to play a role in mediating a wide range of physiological processes, including hormone action, stress response, and cell differentiation [27, 28]. Plant PLDs comprise a large family in their genome; for example, there are 12 members of the PLD family in *A. thaliana*, and expression and functional studies indicate that plant PLDs have functional diversification [29]. In this study, ACC stimulated gene expression of only *NyPLD1*, suggesting that NyPLD1 is responsible for ACC signal transduction, but *NyPLD2, 3* are associated with other cellular process. Previous studies suggest that NyPLD2, 3 may be involved in spore migration and heat stress responses in *N. yezoensis* [20, 21]. Application of a reverse genetic approach has the potential to answer the question why *N. yezoensis* possess three PLDs.

PLD-derived PA has been linked to vesicular trafficking processes including Golgi transport, endocytosis, and exocytosis [30]. This is important, since PA can promote membrane curvature at cisternal rims in the Golgi apparatus, which is required for vesicle formation and budding [31, 32]. For example, 1-butanol treatment decreased the accumulation of PA-mediated secretory vesicles in the apex, resulting in a loss of apical polarity in the pollen tube and inhibited tip growth [33]. Additionally, a previous data suggests that PLD and PA modulate auxin responses through the regulation of vesicle trafficking in *A. thaliana* [34]. In species related to *N. yezoensis*, the active formation of fibrous vesicles has been thought to play a role in protein turnover and cell wall formation during the differentiation of the spermata and carpospores [35]. In addition, our previous study demonstrated that the expression levels of vesicular trafficking-related genes, such as charged multivesicular body protein (CHMP), increased in response to ACC treatment during sexual reproduction in *N. yezoensis* and *P. pseudolinearis* [17]. CHMP proteins are components of the endosomal-sorting complex required for transport (ESCRT) [36], and PLD-produced PA can participate in the assembly of members of the ESCRT machinery in mammal cells [37]. These findings suggest that further research on the involvement of NyPLDs in vesicular trafficking processes may be important in understanding molecular mechanisms involved in red algal reproduction.

ECMs from macroalgae, which are commonly referred to as the cell wall, are complex assemblages of cellulose, various hemicelluloses, and unique sulfated polysaccharides [38]. In this study, 1-butanol treatment alleviated the ACC-downregulation of *NyAly* that was characterized as the gametophyte-specific expressed alginate lyase [39], which may be involved in the change of the composition of the cell wall during *N. yezoensis* life cycle. We also found that 1-butanol inhibited the transcription of ACC- upregulated genes, including ECM-related genes such as *NyGT14*. *NyGT14* is a member of the putative glycosyltransferase family and encodes a homolog of N-acetyl glucosaminyl transferase that can transfer N-acetyl glucosamine (GlcNAc) to an acceptor substrate. Changes in the expression patterns of glycosyltransferase genes have been observed to lead to notable structural alteration of the N-glycans on the cell surface through modification of ECM proteins; this enzyme is thereby associated with various biological events including cell adhesion, migration, and cell differentiation [40]. In addition to *NyGT14*, 1-butanol inhibited the upregulation of two ACC-inducible genes that encode von Willebrand A (VWA) domain-containing proteins. The majority of well-characterized VWA domains are found in ECM proteins, and these domains are involved in protein–protein (e.g., receptor–ligand) interactions [41]. Although little is known about the role ECM proteins play in ACC signaling in *N. yezoensis*, we have also observed that the expression levels of putative ECM genes, including four *NySPL*-encoding spondin domain-containing proteins, were upregulated in response to ACC treatment [42].

Treatment with 1-butanol was found to alleviate transcriptional repression of genes that are downregulated by ACC treatment, including genes related to photosynthesis and *NyPsbQ* and *NySIG*. With respect to photosynthesis, photosystem II (PSII) is the key protein complex involved in light-energy conversion reactions [43]. Moreover, the components of PSII can be classified into core proteins, low-molecular-mass proteins, extrinsic oxygen-evolving complex (OEC) proteins, and the light-harvesting complex II protein [44]. In *Arabidopsis*, the loss of the PsbQ, one of the OEC proteins, induces significant changes in PS II function, particularly in plants grown in low light conditions; this indicates that the PsbQ protein is required for photoautotrophic growth [45]. Regarding the regulation of photosynthesis-related genes, PsbQ is encoded in the nuclear genome, whereas *psbA* and *psbD* encode PS II reaction center core proteins, D1 and D2 polypeptides, and are encoded by plastids [46]. The transcription of plastid genes, including *psbA* and *psbD*, is regulated by nuclear-encoded sigma factors; these bind directly to the core plastid RNA polymerase [47]. In one study in *Arabidopsis*, the authors observed that the transcription levels of *psbA* and *psbD* in SIG1 mutants were significantly lower than those of the wild type [48]. In the present study, the presence of 1-butanol alleviated ACC-induced downregulation of *NySIG*, a gene that encodes a homolog of plant sigma factors. Taken together, the alleviation of the downregulation of photosynthesis-related genes by 1-butanol may help mitigate ACC-induced growth inhibition.

Plant hormones generate ROS through the activation of NADPH oxidase, and this reaction is crucial event in the regulation of plant growth and development [49]. A previous study suggested that ROS generation plays an important role in ACC-induced sexual reproduction in *N. yezoensis* [18]. NADPH oxidases from higher plants possess an extended N-terminus that contain a Ca^2+^ binding EF-hand motif; the activation of these enzymes requires Ca^2+^ binding to the motif [50]. By contrast, red algal NADPH oxidases lack an EF-hand motif which serves as a calcium binding site [51–53], suggesting that red algal NADPH oxidases perceive the calcium signal through other than EF-hand motif [54]. There is evidence to show that plant NADPH oxidases are also directly activated by PLD-derived PA [55], whereas animal NADPH oxidases are indirectly activated through phosphorylation by PA-activated protein kinases [56]. Whether PA stimulates NADPH oxidase from red algae through direct or indirect means or by some more complicated interaction should be investigated in the future.

Plant PLDs are divided into two subclasses. PLDs containing the C2 domain belong to the C2-PLD subclass, whereas those with domains homologous to the domains of phox (PX) and/or pleckstrin (PH) are classified as PX/PH-PLDs [29]. By searching all domains of the protein sequences studied here, we noted that all NyPLDs contained a C2 domain. This makes intuitive sense: This domain is involved in the binding of Ca^2+^ and is known to be required for PLD activation. Previous studies in both plants and animals have demonstrated that ACC can elicit Ca^2+^ currents via glutamate receptors [57–59]. Therefore, ACC-induced Ca^2+^ signaling may play an important role in PLD activation in *N. yezoensis*.

## Conclusion

We demonstrated that increases in PLD activity and PA production follow the accumulation of *NyPLD1* transcripts in response to exogenous ACC treatment. In addition, the pharmacological inhibition of PLD activity alleviated ACC-induced growth repression and blocked ACC-induced formation of spermatangia and carpospores, indicating that PLD plays a negative role for plant growth and a positive role with respect to the differentiation of sex cells. These findings contribute significantly to our understanding of ACC signal transduction during sexual reproduction in red algae.

## Supplementary Information

**Additional file 1: Table S1.** The list of primers used for gene expression analysis by quantitative Real Time PCR. **Table S2.** The list of tested genes for ACC response in *N. yezoensis*.

## Data Availability

All data supporting the findings in this study are presented within the manuscript.
