# Supplementary material for: Phospholipase D activation is required for 1-aminocyclopropane 1-carboxylic acid signaling during sexual reproduction in the marine red alga Neopyropia yezoensis (Rhodophyta)
Source: BMC Plant Biol. 2022 Apr 8;22:181. doi: 10.1186/s12870-022-03575-z (PMC8991923; doi:10.1186/s12870-022-03575-z)
Supplement: Supplementary file 1 — Additional file 1: Table S1. The list of primers used for gene expression analysis by quantitative Real Time PCR. Table S2. The list of tested genes for ACC response in N. yezoensis. [file 12870_2022_3575_MOESM1_ESM.pptx]

## Slide 1
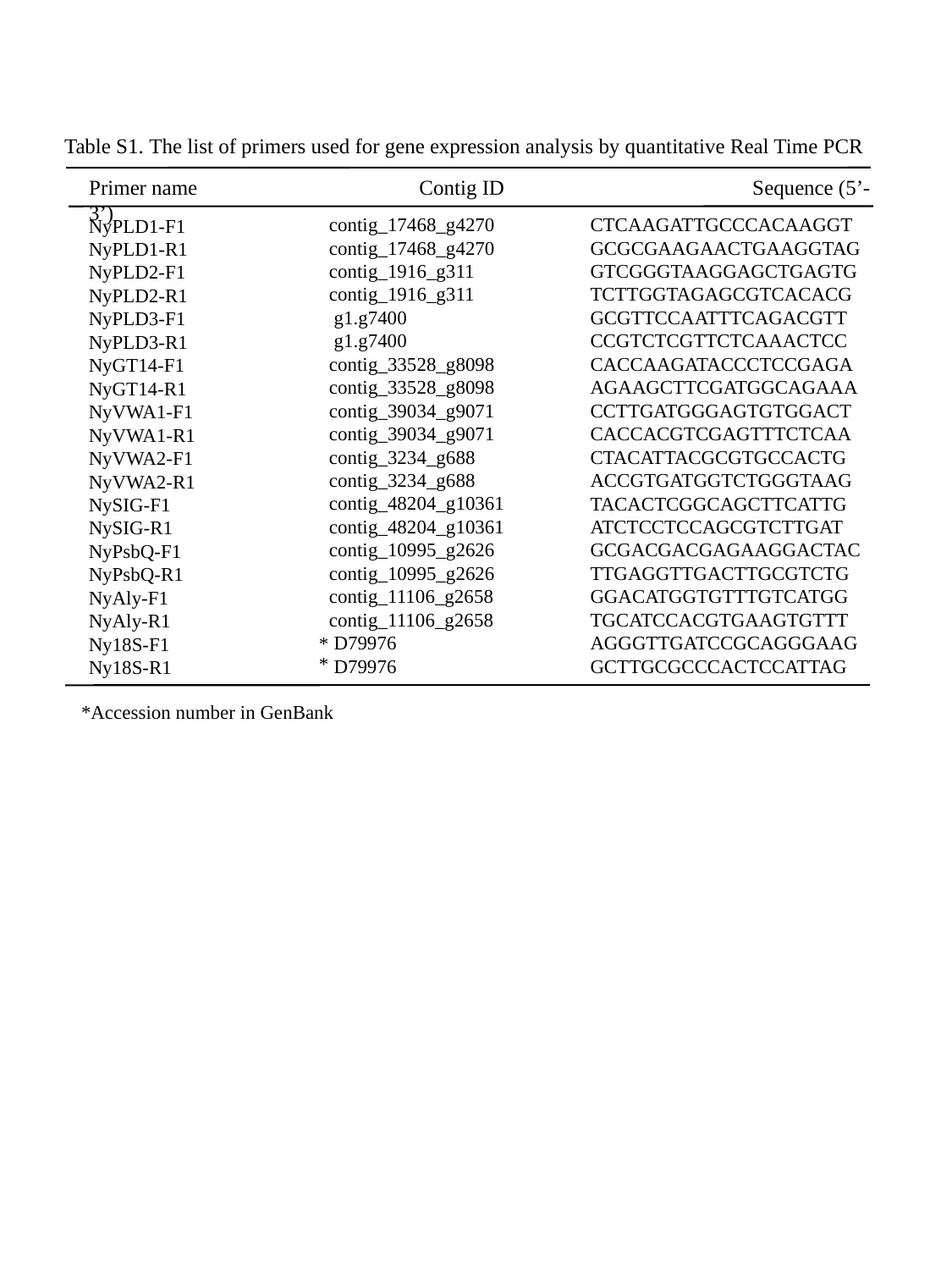

Table S1. The list of primers used for gene expression analysis by quantitative Real Time PCR
Primer name　　　　　　　　　　Contig ID Sequence (5’-3’)
contig_17468_g4270
contig_17468_g4270
contig_1916_g311
contig_1916_g311
 g1.g7400
 g1.g7400
contig_33528_g8098
contig_33528_g8098
contig_39034_g9071
contig_39034_g9071
contig_3234_g688
contig_3234_g688
contig_48204_g10361
contig_48204_g10361
contig_10995_g2626
contig_10995_g2626
contig_11106_g2658
contig_11106_g2658
 D79976
 D79976
CTCAAGATTGCCCACAAGGT
GCGCGAAGAACTGAAGGTAG
GTCGGGTAAGGAGCTGAGTG
TCTTGGTAGAGCGTCACACG
GCGTTCCAATTTCAGACGTT
CCGTCTCGTTCTCAAACTCC
CACCAAGATACCCTCCGAGA
AGAAGCTTCGATGGCAGAAA
CCTTGATGGGAGTGTGGACT
CACCACGTCGAGTTTCTCAA
CTACATTACGCGTGCCACTG
ACCGTGATGGTCTGGGTAAG
TACACTCGGCAGCTTCATTG
ATCTCCTCCAGCGTCTTGAT
GCGACGACGAGAAGGACTAC
TTGAGGTTGACTTGCGTCTG
GGACATGGTGTTTGTCATGG
TGCATCCACGTGAAGTGTTT
AGGGTTGATCCGCAGGGAAG
GCTTGCGCCCACTCCATTAG
NyPLD1-F1
NyPLD1-R1
NyPLD2-F1
NyPLD2-R1
NyPLD3-F1
NyPLD3-R1
NyGT14-F1
NyGT14-R1
NyVWA1-F1
NyVWA1-R1
NyVWA2-F1
NyVWA2-R1
NySIG-F1
NySIG-R1
NyPsbQ-F1
NyPsbQ-R1
NyAly-F1
NyAly-R1
Ny18S-F1
Ny18S-R1
*
*
*Accession number in GenBank

## Slide 2
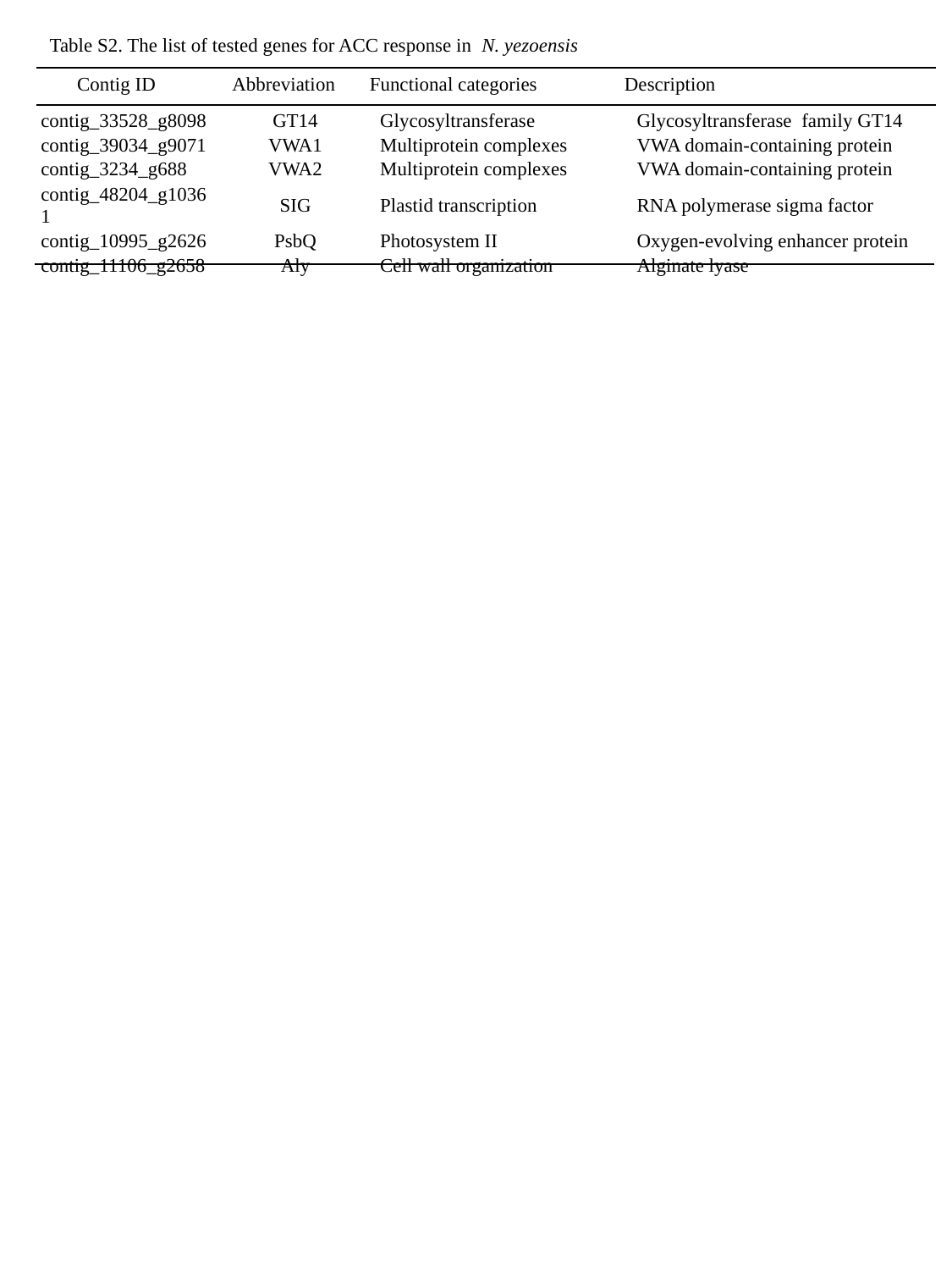

Table S2. The list of tested genes for ACC response in N. yezoensis
 Contig ID Abbreviation Functional categories Description
| contig\_33528\_g8098 | GT14 | Glycosyltransferase | Glycosyltransferase family GT14 |
| --- | --- | --- | --- |
| contig\_39034\_g9071 | VWA1 | Multiprotein complexes | VWA domain-containing protein |
| contig\_3234\_g688 | VWA2 | Multiprotein complexes | VWA domain-containing protein |
| contig\_48204\_g10361 | SIG | Plastid transcription | RNA polymerase sigma factor |
| contig\_10995\_g2626 | PsbQ | Photosystem II | Oxygen-evolving enhancer protein |
| contig\_11106\_g2658 | Aly | Cell wall organization | Alginate lyase |
